# Manipulation and Investigation of Uniformly-Spaced Nanowire Array on a Substrate via Dielectrophoresis and Electrostatic Interaction

**DOI:** 10.3390/nano8070456

**Published:** 2018-06-21

**Authors:** U Hyeok Choi, Ji Hun Park, Jaekyun Kim

**Affiliations:** 1 Department of Polymer Engineering, Pukyong National University, Busan 48547, Korea; uhyeok@pknu.ac.kr; 2 Display Group, R&D Center, IM Co., Ltd., Hwaseong, Gyunggi-Do 18449, Korea; jhpark@im2006.com; 3 Department of Photonics and Nanoelectronics, Hanyang University, Ansan, Gyunggi-Do 15588, Korea

**Keywords:** nanowire spacing, dielectrophoretic force, nanowire-nanowire electrostatic interaction

## Abstract

Directed-assembly of nanowires on the dielectrics-covered parallel electrode structure is capable of producing uniformly-spaced nanowire array at the electrode gap due to dielectrophoretic nanowire attraction and electrostatic nanowire repulsion. Beyond uniformly-spaced nanowire array formation, the control of spacing in the array is beneficial in that it should be the experimental basis of the precise positioning of functional nanowires on a circuit. Here, we investigate the material parameters and bias conditions to modulate the nanowire spacing in the ordered array, where the nanowire array formation is readily attained due to the electrostatic nanowire interaction. A theoretical model for the force calculation and the simulation of the induced charge in the assembled nanowire verifies that the longer nanowires on thicker dielectric layer tend to be assembled with a larger pitch due to the stronger nanowire-nanowire electrostatic repulsion, which is consistent with the experimental results. It was claimed that the stronger dielectrophoretic force is likely to attract more nanowires that are suspended in solution at the electrode gap, causing them to be less-spaced. Thus, we propose a generic mechanism, competition of dielectrophoretic and electrostatic force, to determine the nanowire pitch in an ordered array. Furthermore, this spacing-controlled nanowire array offers a way to fabricate the high-density nanodevice array without nanowire registration.

## 1. Introduction

Nanowires made of a diverse range of chemical compositions, homogeneous or axially and radially heterogeneous, have been demonstrated through various synthetic means [1,2,3,4,5,6,7,8]. Development of novel approaches for producing the parallel array of these nanowires on a substrate or in technological platforms makes it possible to investigate their fundamental properties in an array manner and even to extend its viability to the commercial applications in nanoelectronics, nanophotonics, and nano-biotechnology. To realize this, simple yet sophisticated control of nanowires is required to integrate them on the well-defined region over a large area reliably and reproducibly. Ease of the post-assembly process for the metal contact formation also needs to be considered to assess the potential of assembly scheme [9]. A variety of technological attempts have been made to tackle this fundamental issue of controllable integration of nanowires [10,11,12,13,14,15,16,17]. Despite the successful demonstration of device fabrication in some level, they inherently lack the ultimate control of the final configuration of nanowires, such as the single nanowire deposition, high degree of attraction and orientation of nanowires, excellent end-to-end nanowire registration, and control of nanowire spacing. In order to go beyond the limited success and to meet these controllabilities, we utilized and further refined the dielectrophoresis of nanowires [18,19,20,21]. It was demonstrated elsewhere [9] that the dielectrophoresis of nanowires on the electrode gap of dielectrics-covered interdigitated electrode array is advantageous since (i) single nanowire assembly can be readily attained over large areas due to strong electrostatic repulsion between the nanowires, (ii) >99% nanowire attraction and orientation can be easily achieved by the strong electric field from the electrode gap, (iii) high degree of end-to-end nanowire registration at the predefined position can be achieved by the same length scale of nanowires used, and (iv) the uniformly-spaced nanowire array can be assembled at the electrode gap owing to the repulsive electrostatic interaction between them. Herein, we investigate this assembly scheme in order to fully understand the underlying physics of uniformly-spaced nanowire array formation and vary the spacing of nanowire within the array in a controlled manner, potentially envisioning the registration-free nanodevice array fabrication.

## 2. Experiment Details

For the investigation of nanowire spacing on a substrate, the parallel electrode structure was patterned by a thin metal (Ti/Au = 20 nm/50 nm) following the liftoff process in an acetone dipping. A dielectric layer of polymethylglutarimide (PMGI) with a thickness of 300 nm is covered over the entire substrate so that it prevents the assembly of metallic nanowires from shorting out the biased electrodes. The fluidic cell was used to pull the nanowires in the solution down in the vicinity of the electrically active region. Rhodium (Rh) nanowires were used in this study due to their high elastic modulus, high mechanical strength, and the ease of batch-synthesis via electrodeposition.

After the nanowire suspension is injected into the fluidic cell by using a micropipette, a sinusoidal bias with different voltages and frequencies being applied between the electrodes was applied to investigate the effects of bias conditions on the nanowire attraction to the electrode gap and the nanowire spacing in the array. COMSOL Multiphysics was employed to simulate the electric field distribution and its relevant dielectrophoretic force.

## 3. Results and Discussion

**Figure 1:**
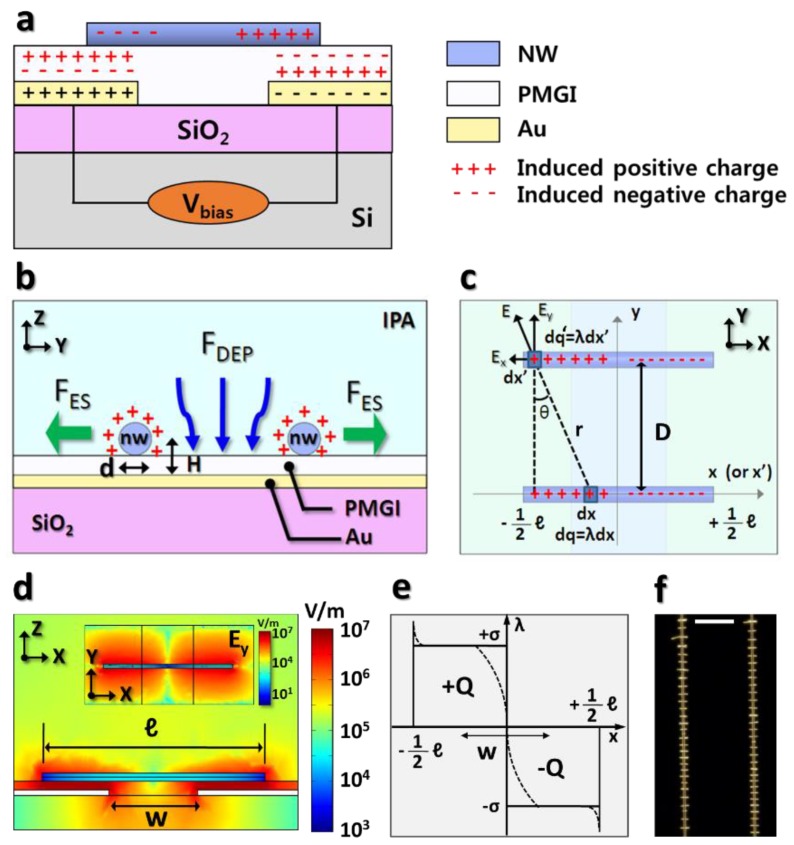
Uniformly-spaced nanowire array formation and its theoretical model. (**a**) A schematic diagram illustrating the induced charges through nanowires due to the PMGI capacitive-coupling to the underlying Au electrodes; (**b**) Schematic illustration of dielectrophoretic nanowire attraction and electrostatic repulsion between the nanowires assembled on the dielectric layer-covered inter-digitated electrode array. The crosses in red on the outer surface of the nanowires represent the induced charges due to their capacitive-coupling to the underlying electrodes; (**c**) Schematic diagram for the theoretical model for the electrostatic interaction between two adjacent nanowires. Double integration of Coulombic force of the charges in the dx’ segment, experienced by the electric field from the charges in dx segment, completes the total force calculation. Taking only the y-component of the total force into account yields nanowires’ repulsive and attractive behavior while bound to the electrode gap; (**d**) Simulation of the electric field at the z-x plane sliced through the nanowire body showing the uniform capacitive-coupling where the nanowire and the biased electrode overlap. The inset exhibits the y-component of the electric field from the polarized nanowires. This also suggests that the density of charges is uniform along the length of the nanowire, except at the electrode gap and the nanowire’s edges; (**e**) Schematic drawing of density of charges induced in the nanowires whose total length is ℓ, assuming that they span the electrode gap symmetrically. Solid and dashed lines refer to the simplified and actual model for the charge distribution, respectively; (**f**) Representative image of 13 μm long rhodium nanowires array on the 3 μm electrode gap covered by PMGI layer, highlighting that they can readily form the uniformly-spaced nanowire array at the electrode gap.

Figure 1a illustrates the cross-section of nanowires alignment structure and induced charge distribution by the capacitive-coupling between the biased electrodes and the assembled metallic nanowires. Biasing to the electrodes enables the induced charge formation in the dielectric layer (PMGI, in this study) as well as in the halves of assembled nanowires with the opposite polarity. These induced charges of nanowires give rise to Coulombic attraction to the substrate during the assembly. Figure 1b describes the dielectrophoretic nanowire attraction and electrostatic repulsion between the assembled nanowires, leading to the uniformly-spaced nanowire formation. The electrostatic repulsive interaction between the assembled nanowires plays a pivotal role in keeping them a few micrometers separated from each other and determining their spacing in the array, while the dielectrophoretic force transports the nanowires toward the electrode gap. Since the assembled metal nanowire locally screens the electric field from the underlying electrode and it has the repulsive force, the additional nanowires are more likely to be assembled in between the existing ones.

The theoretical model accounting for nanowires’ repulsive behavior between two identically polarized nanowires appears in Figure 1c, where the induced charges in the nanowires are labeled “+” for the positive and “−” for the negative charges. The Coulombic interaction between the assembled nanowires involves the repulsion and attraction between them, depending on the polarities of the induced charges within the assembled nanowire. Based on the configuration of two identical nanowires in Figure 1c, the expression for the Coulombic force of the incremental part (dx’) in the nanowire by the electric field from dx is:

(1) $$\;F=\frac{\mathrm{dq}\cdot {\mathrm{dq}}^{\prime }}{4\pi \epsilon_{1}r}$$

where dq and ${\mathrm{dq}}^{\prime }$ are the charges in the nanowires; $\epsilon_{1}$ is the permittivity of the isopropyl alcohol; and, r is the distance between dx and ${\mathrm{dx}}^{\prime }$. Thus, the total Coulombic force, exerted on the assembled nanowire, by the neighboring one can be expressed using double integration of Equation (1) over dx and ${\mathrm{dx}}^{\prime }\;$ from −1/2ℓ to 1/2ℓ

(2) $$\;F_{y}=\frac{1}{4\pi \epsilon_{1}}\int_{-\frac{1}{2}\ell }^{\frac{1}{2}\ell }\left[\int_{-\frac{1}{2}\ell }^{\frac{1}{2}\ell }\frac{\lambda \left(x\right)\cdot y}{{\left({\left(x-x^{\prime }\right)}^{2}+y^{2}\right)}^{\frac{3}{2}}}\mathrm{dx}\right]\lambda \left(x^{\prime }\right)dx^{\prime }$$

where $\epsilon_{1}$ is the permittivity of the isopropyl alcohol; ℓ is the total length of the nanowire; and, λ(x) is the line charge density of nanowires. Here, the effect of the non-uniform charge distribution in the y-direction on the nanowire and any non-uniform charge distribution on the underlying electrodes are neglected. This simplifies the total Coulombic force calculation between the adjacent nanowires, as discussed in the next paragraph.

Due to the charge neutrality, the same amount of positive and negative charges are separated within a nanowire, and their capacitive-coupling to the biased electrodes centers the nanowires at the electrode gap, as illustrated in Figure 1c. The charge distribution on a nanowire also depends on its capacitive-coupling to the underlying electrode. Figure 1d shows the electric field at a cross-section of the z-x plane, sliced through the assembled nanowires where the strong electrostatic interaction arises between the nanowires and the biased electrodes. Because the induced charge relates to this electrostatic interaction, its density along the nanowire length can be depicted as the dashed line in Figure 1e.

Theoretically, it can be suggested from Figure 1c that the charges uniformly distribute on the nanowire where the nanowire and the biased electrode overlap, except at the edges and the central region of a nanowire because of electric field condensation and charge transition, respectively. The inset in Figure 1d exhibits the y-component of the nanowires’ electric fields, which causes the assembled nanowires repel each other. As a result, Figure 1f shows the dark-field optical microscope image of a 7 μm long Rh nanowire array with a uniform spacing being assembled at the electrode gap.

An approximation suggests that the induced charge density is uniform on either half of the nanowire with an opposite sign of the charge density. This approximation, for the simple model, as drawn as a solid line in Figure 1e, can be expressed:

(3) $$\;\lambda \left(x\right)=+\sigma \;\mathrm{for}-\frac{1}{2}\;\ell \leq x\leq 0,\;\mathrm{and}$$

(4) $$\;\lambda \left(x\right)=-\sigma \;\mathrm{for}\;0\leq x\leq +\frac{1}{2}\;\ell .$$

Using this simple uniform charge density model, the total Coulombic interaction force, F_y_, between the assembled nanowires can be written:

(5) $$\;F_{y}=\frac{2\sigma^{2}}{4\pi \epsilon_{1}}\left[2\left\{\sqrt{1+\frac{1}{4}{\left(\frac{\ell }{y}\right)}^{2}}-1\right\}-\;\left\{\sqrt{1+{\left(\frac{\ell }{y}\right)}^{2}}-\;2\sqrt{1+\frac{1}{4}{\left(\frac{\ell }{y}\right)}^{2}}+1\right\}\right]$$

The first term in the equation refers to the repulsive force between two adjacent nanowires, whereas the second term refers to the attractive force. Since the electrostatic interaction between the nearest neighboring nanowires is dominant, this total force calculation can readily apply to all of the nanowires assembled at the electrode gap. Equation (5) suggests that the total force is always positive in any case and is inversely proportional to the distance between the adjacent nanowires when they are close. This condition results in strong repulsive force at small distances. 

**Figure 2:**
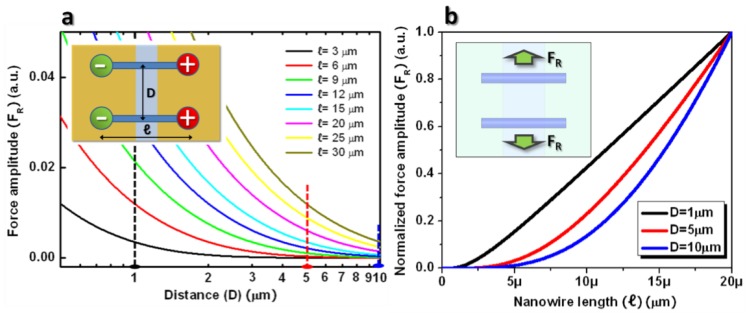
Inter-nanowire interaction force as a function of nanowire distance and length. (**a**) Plot for the relation between the force, F_y_, and the nanowire spacing, y, while varying the nanowire length, ℓ, from 3 μm to 30 μm. The positive value in the plot refers to the repulsive force between the nanowires, indicating that they tend to be spaced at all different lengths of nanowires. The vertical dashed lines are drawn to compare the forces at the constant distances. The inset illustrates dumbbell dipole structure for electrostatic nanowire interaction; (**b**) Plot of the normalized force as a function of a nanowire’s length, ℓ, at fixed nanowire distance, y = 1, 5, and 10 μm, marked by the dashed lines in (**a**). The inset illustrates a nanowire’s repulsion, F_R_, at the electrode gap.

Plotting Equation (5) provides more useful information to clarify the relationship between the repulsive nanowire interaction and nanowires’ material parameters, such as the inter-nanowire distance and the nanowire length, as shown in Figure 2a, displaying the total Coulombic interaction force, F_y_, (Equation (5)) as a function of the assembled nanowire distance (y) at various nanowires’ lengths (ℓ). This plot predicts that the repulsive force (F_R_) tends to become less strong as the inter-nanowires’ separation distances (D) increase. A further approximation from the previous model indicates that the uniform charge density of half of the assembled nanowires can easily clarify this tendency. In other words, the polarized nanowires can be a further approximation of dumbbell dipole structures having the opposite charges, positive and negative, at the ends of the dumbbells, as shown in the inset of Figure 2a. Simply, two forces exist: i.e., the repulsive force between the charges with the same polarities and the attractive force with the different polarities. At very small distances (D) between the dumbbells, the repulsive forces dominate over the attractive forces, since the distances between the same polarities are much smaller than the different polarities. This strong repulsion keeps the assembled nanowires a few micrometers apart, leading to a single nanowire assembly. If the inter-nanowire distance (D) becomes much larger, compared to a nanowire’s length (ℓ), the repulsive force tends to converge as the attractive force while each force is still in effect. As a result, the overall electrostatic interaction between the assembled nanowires becomes nearly negligible, making them static between the biased electrodes. Consistent with this intuitive explanation, Figure 2a shows that the force amplitude indicates a positive value, namely repulsive force, while this force amplitude tends to converge at zero at larger inter-nanowire spacing when compared to nanowire length.

The uniformly-spaced nanowire array begins to form as the nanowire spacing comes close to a certain value for efficient electrostatic interaction. According to the plot in Figure 2a, this tendency becomes more significant in the longer nanowires, hinting that the nanowires can easily form a uniformly-spaced nanowire array. It is also easily recognized from the plot that the longer nanowires experience a higher degree of repulsion between them at the same inter-nanowire distance. This relation is intuitively sensible because longer nanowires have more overlapping by the underlying biased electrode, leading to higher repulsion between the assembled nanowires, which is primarily due to more charges within them. Additionally, the repulsive forces are normalized by the maximum values of different nanowire lengths, whose functions are plotted in Figure 2b to investigate their relationships at the fixed nanowire spacing. This plot reveals that the repulsive force develops into an approximately linear relationship with the lengths of nanowires (ℓ), particularly at small nanowire spacing (D). This force relates to the average spacing within the nanowire array.

**Figure 3:**
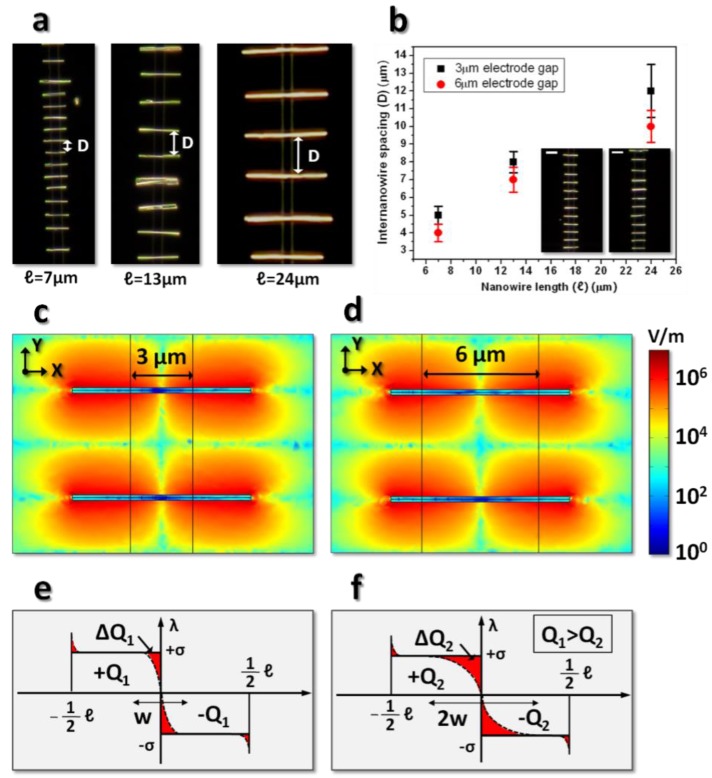
Inter-nanowire spacing as a function of the nanowire length and electrode gap width. (**a**) Representative dark-field optical microscope images of an array with different nanowire lengths (ℓ = 7, 13, and 24 μm), also indicating the different inter-nanowire spacing, proportional to their lengths. (**b**) Plot for the inter-nanowire distance with different nanowire lengths (ℓ = 7, 13, and 24 μm) where different widths of electrode gaps, 3 μm (black square) and 6 μm (red circle), are employed. This plot shows that the nanowires tend to be less spaced with wider electrode gaps, while forming the ordered array. The inset exhibits a 7 μm long rhodium nanowire array with 3 μm (left) and 6 μm (right) electrode gaps. The simulation of a y-component electric field, E_y_, on the nanowires assembled with (**c**) 3 μm and (**d**) 6 μm wide electrode gaps. Schematic drawing of the charge density along a nanowire’s length, highlighting the effect of (**e**) 3 μm and (**f**) 6 μm wide electrode gaps. Noticeably, the central part of nanowires’ overlapping the electrode gap will induce a lower charge density (λ) and the electric field (E_y_) of the nanowires, eventually affecting the nanowires’ spacing.

Nanowire assembly, accomplished with the same interdigitated electrode structure varied nanowire length (7, 13, and 24 μm long rhodium nanowires). For consistency of the nanowire assembly, the concentration of the nanowire suspensions remained as low as ~1 × 10^4^ nanowires/μL by adding isopropyl alcohol. Special attention maintained injection of 10 μL of nanowire suspension for all of the length scales until the excessive nanowire-nanowire chain configuration began to form. Figure 3a shows dark-field optical microscope images of 7, 13, and 24 μm long rhodium nanowires in 3 μm spaced electrode gaps. Note that non-uniformity of nanomembranes used for the electrodeposition of rhodium nanowires yielded distribution of nanowire diameter and shape. Consistent with the theoretical expectation in Equation (5), serial images indicate that the longer nanowires seem to have larger spacing between them, while they sustain the integrity of an ordered nanowire array. Furthermore, nanowire spacing at different nanowire lengths form the plot in Figure 3b where the average inter-nanowire spacing increases almost linearly as the nanowire length increases. This linear relationship between the nanowire spacing and length seems to coincide with the plot for the force calculation of the nanowires with different lengths, as indicated in Figure 2b.

Figure 3b also shows the nanowires’ assembly results with a 6 μm-spaced electrode gap with the effect of different electrode gaps’ widths on the nanowire spacing. The insets show the image of a 7 μm-long nanowire assembly with 3 μm (left) and 6 μm (right) electrode gaps. Obvious from the comparison between 3 μm and 6 μm electrode gaps in the plot of Figure 3b is that the same lengths of nanowires with the wider electrode gap become slightly less spaced. Recalling the uniform charge distribution model for the assembled nanowires earlier explains the influence of electrode gap width on nanowire spacing.

In principle, the charge density begins to drop as it passes the edge of an underlying electrode and it falls to zero at the exact middle of nanowires, symmetrically assembled at the electrode gap. The charge density remains constant at locations that are away from the mid and the edge areas. The simulated E_y_ distribution for 3 μm and 6 μm wide electrode gaps in Figure 3c,d, respectively, shows that E_y_ becomes less strong beyond the edge of a biased electrode, particularly in a narrow electrode gap. According to Gauss’s law ($\oint^{\text{​}}\;\vec{E}\cdot d\;\vec{A}=Q/\epsilon_{0}$), distribution emerging from the nanowire allows for an indirect estimate of the charge distribution of the assembled nanowires.

As a result, the charge density can be drawn in a way that the nanowire assembled on a wide electrode gap has fewer charges when compared to the narrow one, as described in Figure 3e,f. ΔQ_1_ and ΔQ_2_ in Figure 3e,f, respectively, refer to the differences of the charges of the simple model with a step function-like uniform charge density. Thus, the Coulombic force between the nanowires becomes larger for the narrow electrode gap, leading to slightly larger nanowire spacing, as shown in Figure 3b.

Again, the approach that uses the dielectrophoretic nanowire attraction and electrostatic nanowire interaction yields an ordered nanowire alignment at the electrode gap with a uniform spacing. Other methods have demonstrated an ability to define the average spacing between the assembled nanowires, using a lithographically patterned surface [12], a surface functionalization of the substrate [11], a concentration of nanowires in the suspension [16], and a compression process [22]. The nanowire array with average nanowire spacing provides an opportunity to pursue registration-free, single nanowire device fabrication on an array platform [22,23], where success relies on the precise control of nanowire spacing with small fluctuations in local scale.

According to Equation (5), the electrostatic repulsive force can be determined by the value of the charge density (σ) on the assembled nanowires. Approximation of the nanowire-electrode system to the parallel capacitor leads to modulation of the induced charges in the polarized nanowires as a function of bias conditions and the materials’ parameters in the alignment structure, all of which eventually affect nanowire spacing. The charge (Q) of the nanowire using a simple capacitor model and the capacitance (C) of the nanowire on the biased electrode approximation has the expression [24].

(6) $$\;Q\;=\;C\;\cdot \;V\;=\;\frac{\pi \epsilon \ell }{\cosh^{-1}\left(2H/d\right)}V$$

where ϵ  is the permittivity of dielectric layer; H is the distance between the biased electrode and the center of nanowire; d is the diameter of nanowire; and, V is the potential between the nanowire and the electrode. When fixing the nanowire length and diameter at a constant value through batch fabrication, the amount of charges in the nanowires depends on the amplitude of applied bias and the thickness of the dielectric layer.

The frequency of the applied bias could influence nanowire alignment on a biased electrode. In other words, the frequency response of dipole moment in the polarized nanowires determines the dielectrophoretic nanowire attraction by the real part of the Clausius-Mossotti factor [R_e_(K)]. The frequency of applied bias also affects the induced charges in the assembled nanowires, resulting in the frequency-dependent electrostatic force between them. In contrast to the dipole formation in semiconducting nanowires, the metal is of a very high permittivity material due to free electrons in it. Ideally, an infinite value makes R_e_(K) almost constant over a wide range of frequencies. Without doubt, the frequency response of free electrons in the metal is instant. Thus, the induced charges in the assembled nanowires as well as the dielectrophoretic attraction of them can be considered as frequency-independent factors for determining the nanowires’ spacing in the array. The average spacing of 7 μm-long rhodium nanowires, assembled at the electrode gap with a 3 μm width, is estimated to be about 5 μm in the range of 10 kHz to 10 MHz. As discussed, no significant change in the nanowire spacing was observed at relatively low frequency ranges, as compared to the relaxation time of free electrons in the metal.

**Figure 4:**
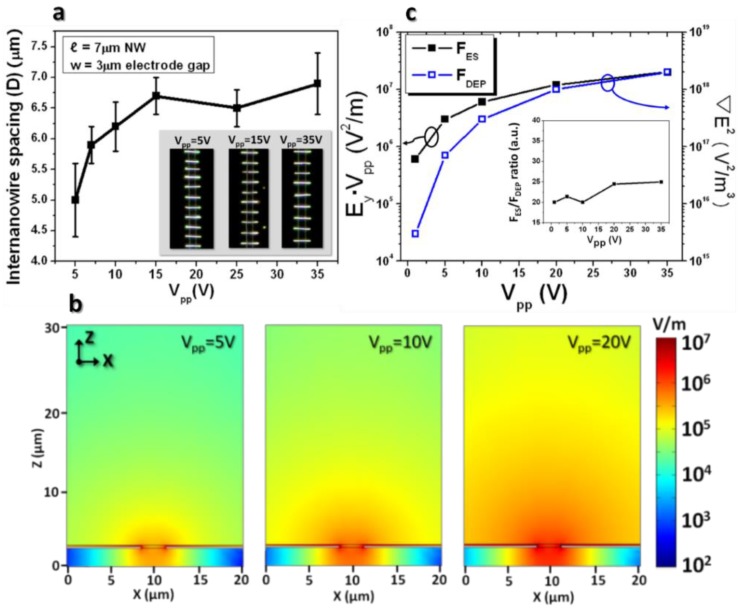
Inter-nanowire spacing as a function of the applied bias. (**a**) Plot of average nanowire spacing as a function of the voltage of applied bias, V_pp_. Apparently, the inter-nanowire spacing increases gradually as the voltage of applied bias ramps up from 5 V to 35 V. Dark field optical microscope images of the nanowire array are obtained from V_pp_ = 5, 15, and 35 V. Scale bar = 5 μm. (**b**) Normalized electric field simulation at different voltages of applied bias (V_pp_ = 5, 10, and 20 V) exhibiting the electric field’s spread toward the liquid medium. The nanowires will be polarized and dielectrophoretically attracted toward the electrode gap if they are within the decay length of the critical electric field. (**c**) Plot of the electrostatic repulsive E_y_·V_pp_ and dielectrophoretic $\nabla E^{2}$ forces as functions of the applied bias strength. The inset emphasizes that ratios of the electrostatic to dielectrophoretic forces increase relatively gradually as the applied bias increases.

Figure 4a shows the plot for nanowires’ spacing (D) as a function of the peak-to-peak voltage of applied bias (V_pp_). It was observed that the 7 μm-long rhodium nanowires form uniformly-spaced arrays on the electrode gap for all of the voltage ranges. In contrast to the theoretical expectation that is described in Equations (5) and (6), the plot (Figure 4a) demonstrates that the nanowire spacing tends to increases gradually as the bias voltage ramps up. This discrepancy might appear intuitively inapprehensible until the effect of voltage for trapping the nanowires from the suspension is taken into account.

In order to investigate the dependence of the applied voltage for the nanowire assembly, visualization of the spatial distribution of the electric field from the electrode gap can be beneficial. Figure 4b displays the spatial electric field distributions of the z-x planes toward the nanowire solution at V_pp_ = 5, 10, and 20 V. Comparison of the spatial electric field distributions as a function of amplitude of applied bias clearly contrasts the critical nature of field strength spread in the suspension with almost linearly proportionality of the applied voltage. For example, predictably from Figure 4b, the minimum electric field that is required for nanowire attraction, namely about 10^6^ V/m, at V_pp_ = 10 V, extends toward the nanowire suspension two times further than at V_pp_ = 5 V in the nanowire assembly. This tendency suggests that more nanowires in the suspension are within an electric field of effective nanowire polarization at higher voltages, resulting in a nanowire array formation with less spacing.

Comparison of the dielectrophoretic force (F_DEP_), which is responsible for the number of nanowires to be assembled, and the electrostatic repulsion (F_ES_) between the nanowires, responsible for the inter-nanowire spacing, is a reasonable indication for determining the inter-nanowire spacing for given conditions. The expression of the dielectrophoretic force is [25].

(7)  FDEP = πd2ℓ24 ε1 Re (K) ∇E2

(8) $$\;F_{\mathrm{ES}}\;=\;\left(\frac{2\pi \epsilon }{\cosh^{-1}\left(2H/d\right)}\frac{\ell }{2}V\right)\cdot E_{y}$$

where $\varepsilon_{1}$ is the permittivity of the liquid medium, E is the applied electric filed, and Re(K) is the real part of the Clausius-Mossotti factor. Using the simulation, the ratio of the electrostatic repulsive (F_ES_) to the dielectrophoretic (F_DEP_) forces enables the estimation of the relative inter-nanowire spacing in the array according to the chosen specific bias conditions and materials’ parameters. In other words, their high ratios of electrostatic to dielectrophoretic forces at the given conditions of applied voltage provide an indication of the relatively stronger electrostatic force between the assembled nanowires compared to the dielectrophoretic force, leading to larger spacing between the nanowires.

Figure 4c shows the plot of the ratio of the electrostatic (F_ES_) and dielectrophoretic (F_DEP_) forces in which E_y_ for F_ES_ and $\nabla E^{2}$ for F_DEP_ are extracted from the PMGI surface above the electrode edge and the surface of the assembled nanowires in the simulations, respectively. The voltage varied from 1 V_pp_ to 35 V_pp_. As anticipated from the equations, notably, from Figure 4a, the electrostatic repulsive force and the dielectrophoretic force increase as the voltage ramps up. As explained earlier in this paragraph, comparison of the ratios is more important for clarifying the relationship between the inter-nanowire spacing and the voltage. The inset in Figure 4c shows that the ratio of F_ES_/F_DEP_ increases by a small increment as the voltage increases. This further suggests that the inter-nanowire spacing at varying voltages has the same relationship. Apparently, this relationship is quite consistent with the plot of the experimental results that are shown in Figure 4a.

**Figure 5:**
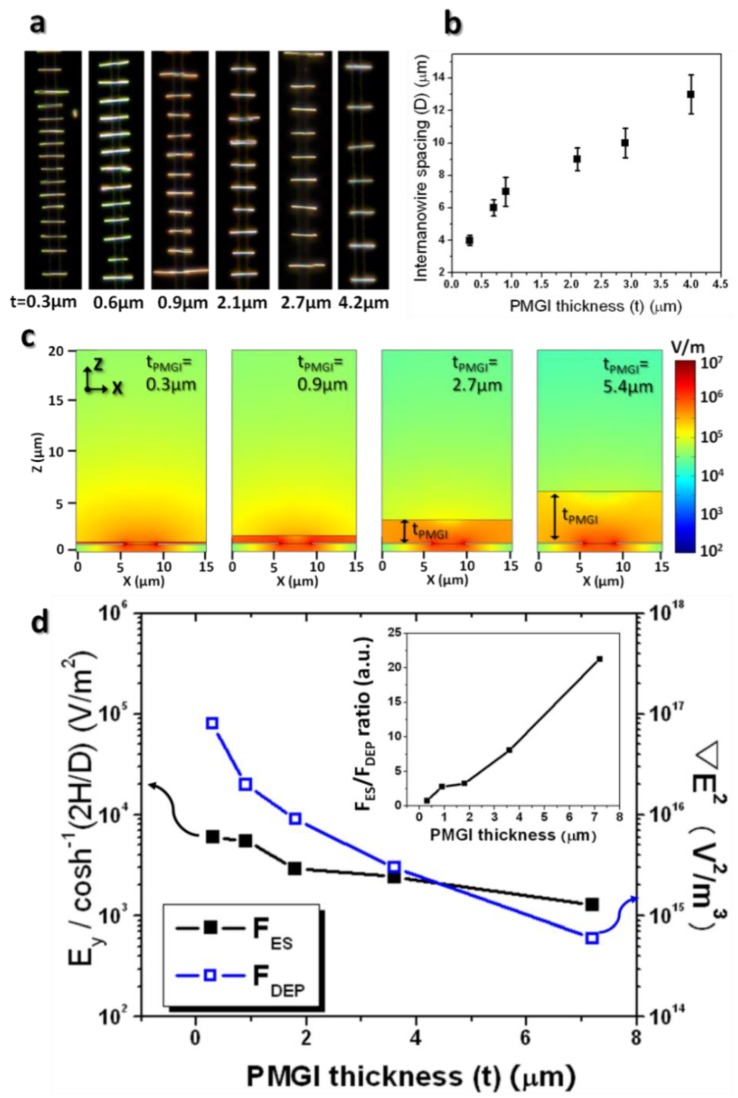
Inter-nanowire spacing as a function of the dielectric layer thickness. (**a**) Series of dark-field optical microscopic images for the different thicknesses of PMGI layers (t_PMGI_ = 0.3, 0.6, 0.9, 2.1, 2.7, and 4.2 μm), and (**b**) its plot for the relationship between the inter-nanowire spacing and PMGI thicknesses. (**c**) Spatial electric field distribution at different thicknesses of PMGI layers (t_PMGI_ = 0.3, 0.9, 2.7, and 5.4 μm) exhibiting the electric field’s spread toward the liquid medium. Smaller number of nanowires in the suspension will be polarized, dielectrophoretically attracted, and redistributed on thicker PMGI layers, leading to smaller inter-nanowire spacing. This is due to the electric field’s decaying relatively quickly on thick PMGI layers. (**d**) Plot of the electrostatic repulsive (E_y_/cosh^−1^(2H/D)) and dielectrophoretic ($\nabla E^{2}$) forces as functions of PMGI thickness. The inset shows that ratios of electrostatic to dielectrophoretic forces increase nearly linearly as the PMGI thickness increases.

The thickness of dielectric layer, i.e., PMGI in the experiment’s assembly setup, also affects the electrostatic repulsion between the assembled nanowires, eventually establishing the average spacing between them. Understandably, the amount of induced charge on the surface of the nanowires depends on the thickness of the dielectric layer because of the capacitor-like structure of nanowire-PMGI-biased electrodes [26]. Figure 5a exhibits the serial images of assembly results while varying the PMGI thickness (t), explicitly displaying the average spacing in the nanowire array, which is virtually free of the chain formation, and becoming larger as the dielectric layer increases. The plot in Figure 5b illustrates that the inter-nanowire spacing is almost linearly proportional to the PMGI thickness. These results indicate that the electrostatic interaction of nanowires still holds its effect for the uniform spacing with an array in the case of a PMGI layer of about 4 μm. Figure 5c clearly shows that the electric field in the suspension as well as in the vicinity of the electrode gap becomes weaker with a thicker PMGI layer that is spun on it. Evident from Figure 5c is that the electric field at the electrode gap decreases by nearly two orders of magnitude as the thickness of the PMGI layer decreases from 0.3 μm to 5.4 μm.

In order to interpret this inter-nanowire spacing dependence on the thickness of PMGI, performing a simulation to compare the ratio of the electrostatic (F_ES_) and dielectrophoretic (F_DEP_) forces is necessary. Similarly, a plot of E_y_/cosh^−1^(2H/D) for the inter-nanowire repulsion force and $\nabla E^{2}$ for the dielectrophoretic force as functions of PMGI thickness from 0.3 μm to 7.2 μm appears in Figure 5d. This plot describes that both forces at the surface of the PMGI layer diminish as PMGI thickness increases. This relationship is compatible with the theoretical expectation from Equations (7) and (8). The inset of Figure 5d, however, shows that their ratios of F_ES_ to F_DEP_ increase nearly linearly as PMGI thickness increases, suggesting that the electrostatic force between the assembled nanowires becomes relatively stronger when compared to the dielectrophoretic nanowire attraction. Thus, the ratios of E_y_/cosh^−1^(2H/D) to $\nabla E^{2}$ seem to provide an adequate account for the relationship between the electrostatic and dielectrophoretic forces, namely sensitive inter-nanowire spacing change according to differing PMGI thicknesses. Worthy of note is that strong capacitive-coupling of thin PMGI layers might encounter relatively strong friction, when compared to thick PMGI layers that resist electrostatic repulsion motion. This friction also can contribute to larger spacing between the assembled nanowires on thick PMGI layers.

## 4. Conclusions

This study of control of the inter-nanowire spacing in the array demonstrated that the electrostatic repulsion between the nanowires plays a key role in determining the average distance between them, while the dielectrophoretic force also contributes to it by controlling the number of nanowires to be assembled. This electrostatic repulsion as well as the dielectrophoretic force is not only limited to the metallic entities and is also applicable to other semiconducting nanowires. Significance of this study lies on the fact that the average spacing between the nanowires can be readily controlled either by the bias conditions or by the materials parameters. In addition to the unique feature of our nanowire assembly, such as a high degree of alignment and orientation at well-defined locations and excellent end-to-end registration, this control of nanowires spacing offers a promising method without the need for the registration of nanowires to fabricate the individual devices by the metal deposition following the lithographical patterning. For instance, setting the width of narrow metal interconnects to the average distance of the nanowires produces a large number of single functional nanowire devices in a scalable and parallel fashion where the nanowire registration relative to the metal electrode is not required. Its versatility could be extended to the novel applications, such as the plasmonics of metallic nanopattern and nanoresonator devices, chemical and biological sensors, and flexible light-emitting diode, where the multiple nanowires with a tight control of its number are preferred for the uniform transistors array requiring high transconductance.
